# Neuropsychological Sub‐Phenotypes in Amyotrophic Lateral Sclerosis

**DOI:** 10.1111/ene.70706

**Published:** 2026-08-03

**Authors:** Barbara Poletti, Edoardo Nicolò Aiello, Monica Consonni, Barbara Iazzolino, Silvia Torre, Veronica Faltracco, Alessandra Telesca, Francesca Palumbo, Beatrice Curti, Giulia De Luca, Arianna Moreschi, Francesca Frisco, Eleonora Dalla Bella, Enrica Bersano, Nilo Riva, Federico Verde, Stefano Messina, Alberto Doretti, Alessio Maranzano, Claudia Morelli, Stefano Francesco Cappa, Andrea Calvo, Michael J. Strong, Vincenzo Silani, Giuseppe Lauria, Adriano Chiò, Nicola Ticozzi

**Affiliations:** ^1^ Department of Neurology and Laboratory of Neuroscience IRCCS Istituto Auxologico Italiano Milano Italy; ^2^ Department of Oncology and Hemato‐Oncology Università Degli Studi di Milano Milano Italy; ^3^ 3rd Neurology Unit and Motor Neuron Disease Centre Fondazione IRCCS Istituto Neurologico Carlo Besta Milan Italy; ^4^ “Rita Levi Montalcini' Department of Neuroscience, Amyotrophic Lateral Sclerosis Center University of Turin Torino Italy; ^5^ Neuroalgology Unit, Department of Clinical Neuroscience Fondazione IRCCS Istituto Neurologico Carlo Besta Milan Italy; ^6^ Neurointensive Care Unit, Department of Neurosurgery Fondazione IRCCS Istituto Neurologico Carlo Besta Milan Italy; ^7^ Department of Medical Biotechnology and Translational Medicine Università Degli Studi di Milano Milan Italy; ^8^ Neurology 1 City of Health and Science University Hospital of Turin Torino Italy; ^9^ Molecular Medicine Group, Robarts Research Institute, Schulich School of Medicine and Dentistry Western University London Ontario Canada; ^10^ Department of Pathology and Laboratory Medicine, Schulich School of Medicine and Dentistry Western University London Ontario Canada; ^11^ Department of Clinical Neurological Sciences, Schulich School of Medicine and Dentistry Western University London Ontario Canada; ^12^ Department of Pathophysiology and Transplantation, Dino Ferrari Center Università Degli Studi di Milano Milano Italy; ^13^ Institute of Cognitive Science and Technologies National Research Council Roma Italy

**Keywords:** amyotrophic lateral sclerosis, edinburgh cognitive and behavioral ALS screen, frontotemporal‐*spectrum* disorder, neuropsychology, phenotype, research criteria

## Abstract

**Background:**

This study aimed at identifying neuropsychological sub‐phenotypes in amyotrophic lateral sclerosis (ALS) within the mild cognitive impairment (MCI) and mild behavioral impairment (MBI) frameworks.

**Methods:**

We used individual task−/item‐level data from the cognitive and behavioral sections of the Edinburgh Cognitive and Behavioral ALS Screen (ECAS) from 901 non‐demented ALS to derive neuropsychological sub‐phenotypes pursuant to classical MCI and MBI frameworks and in accordance with an expanded version of Strong's criteria, which also addressed memory and visuo‐spatial measures.

**Results:**

The prevalence of MCI and MBI was 39% and 37%, respectively in this retrospective review. The following MCI sub‐phenotypes were identified: dysexecutive MCI—single‐ and multiple‐domain (dMCI‐sd: 63%; dMCI‐md: 24%, respectively); non‐dysexecutive MCI—single‐ and multiple‐domain (ndMCI‐sd: 12%; ndMCI‐md: 1%, respectively). MBI was classified as follows: apathetic MBI—single‐ and multiple‐domain (aMBI‐sd: 40%; aMBI‐md: 20%, respectively); apathetic‐disinihibited/perseverative MBI—multiple domain (ad/pMBI‐md: 21%); disinihibited/perseverative MBI—multiple domain (d/pMBI‐md: 7%); psychotic MBI—single‐ and multiple‐domain (psyMBI‐sd: 2%; psyMBI‐md: 3%, respectively); unclassifiable MBI—multiple domain (uMBI‐md: 1%). 143 (16%) of patients exhibited mild cognitive and behavioral impairment (MCBI).

**Conclusions:**

This study delivers a provisional, ECAS‐based classification for the neuropsychological sub‐phenotyping of non‐demented ALS patients, which, with further validation, might be useful for both research and clinical purposes.

## Graphical abstract

Evaluating 901 non‐demented ALS patients with the ECAS (Edinburgh Cognitive and Behavioral ALS Screen), this study proposes a classification of neuropsychological sub‐phenotypes based on mild cognitive and behavioral impairment (MCI/MBI) profiles. MCI and MBI were observed in 39% and 37% of patients, respectively, with dysexecutive MCI and apathetic or apathetic‐disinhibited/perseverative MBI representing the most frequent profiles; 16% showed a combined MCI/MBI profile. This stratification of single‐ and multiple‐domain deficits may improve patient characterization, clinical monitoring, and research stratification.

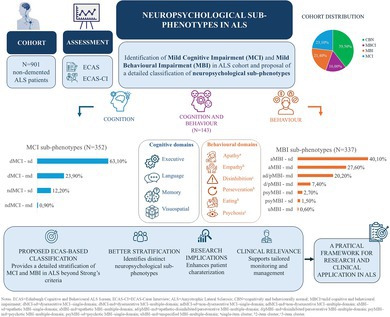

## 1 Background

The current nosographic system for mild neuropsychological disorders in amyotrophic lateral sclerosis (ALS) by Strong et al. [1] defines mild cognitive impairment (MCI) as the occurrence of either executive dysfunctions or language deficits and mild behavioral impairment (MBI) as the presence of either apathy alone and/or of at least two other behavioral changes typical of behavioral variant‐frontotemporal dementia. However, such a classification does not allow for patient neuropsychological sub‐phenotyping—i.e., to determine whether patients present with single‐ vs. multiple‐domain MCI/MBI, as well as to profile them according to their specific impairment patterns.

Moreover, there is growing evidence suggesting that the range of possible cognitive disturbances is wider than previously thought [2, 3, 4]. For instance, memory and visuo‐spatial deficits [5], once thought to be atypical of ALS [6], are increasingly being observed.

To address these issues, Spisto et al. [7] have proposed a complementary nosographic system—the Mild Behavioral and Neurocognitive Impairment (MBNI) approach—relying on the frameworks of MCI [8] and MBI [9] for other neurodegenerative conditions. These authors showed that, when applying the MBNI, a number of patients classified as free of neuropsychological changes under Strong's criteria could be categorized as having some degree of cognitive and/or behavioral alterations. Most relevantly, Spisto et al. [7] also showed that cognitive and/or behavioral disturbances left undetected by Strong's criteria detrimentally impacted patients' disease trajectories—thus pointing out that a more comprehensive and accurate characterization of ALS patients' neuropsychological changes might also come with relevant prognostic entailments.

With that being said, whilst having the merit of also including measures of so‐called “ALS‐nonspecific” cognitive domains (i.e., memory and visuospatial abilities), the MBNI is limited in generalizability because of the use of second‐level cognitive measures that did not compensate for ALS patients' verbal‐motor disabilities. Moreover, despite including a large cohort (*N* = 201), Spisto and colleagues did not provide insights into patients' specific neuropsychological sub‐phenotypes—possibly because the frequency of patients falling under each individual category was relatively limited in number.

In order to grant a sufficient degree of generalizability, a proposal for identifying neuropsychological sub‐phenotypes in ALS based on “classical” MCI and MBI frameworks should include a sufficiently large sample size and employ gold‐standard instruments that compensate for patients' verbal‐motor disabilities—such as the Edinburgh Cognitive and Behavioral ALS Screen (ECAS) [10].

Hence, the present study aimed at identifying neuropsychological sub‐phenotypes for MCI and MBI in ALS by addressing a large (*N* = 901) cohort of non‐demented patients assessed via the ECAS, as well as at preliminarily providing an ECAS‐based set of criteria for the classification of mild cognitive and/or behavioral disturbances in this population.

## 2 Methods

### 2.1 Participants

This retrospective cohort comprised 901 ALS patients without a concurrent clinical diagnosis of frontotemporal dementia [11, 12] drawn from three Northern Italian centers (i.e., IRCCS Istituto Auxologico Italiano, Milano; Fondazione IRCCS Istituto Neurologico Carlo Besta, Milano; City of Health and Science University Hospital of Turin, Torino) using a convenience sampling technique between 2016 and 2023 and for whom the cognitive sections of the ECAS [13] and the ECAS‐Carer Interview (ECAS‐CI) [14] were available.

### 2.2 Materials

Patients MCI/MBI classifications were derived by addressing individual tasks from the cognitive section of the ECAS and individual items from the ECAS‐CI. Cognitive screening and semi‐structured interviews with patients' carers on behavioral changes were conducted by senior ALS neuropsychologists who underwent standardized training for ECAS administration and scoring. As to MCI, in accordance with Petersen [8], a first subdivision was made between single‐ and multiple‐domain MCI. The same subdivision was then applied to MBI.

Cognitive impairment within a given domain was defined as previously published [15]. Accordingly, executive dysfunctions were defined on the basis of either ≥ 1 below‐cutoff subtest from the ECAS‐Fluency or ≥ 2 below cutoff subtests from the ECAS‐Executive, while, as to language, on the basis of ≥ 2 below cutoff subtests from the ECAS‐Language. With respect to the memory and visuospatial domains, a similar approach was followed, with memory impairment being defined by ≥ 2 below‐cutoff subtests from the ECAS‐Memory and visuospatial impairment by ≥ 2 below‐cutoff subtests from the ECAS‐Visuospatial.

Behavioral impairment was defined based on ECAS‐CI scores as previously described by Poletti and colleagues [15]. Consistent with the Strong criteria, ALSbi was defined as either the presence of apathy, with or without other behavioral changes, or at least 2 positive symptom clusters from the ECAS‐CI. We also considered the Psychosis symptom cluster, which is not considered within Strong's criteria for this category. A positive finding in the Psychosis cluster was defined as the presence of at least one of its items.

In defining patients' MCI/MBI classifications, priority was given to executive deficits and apathy when addressing MCI and MBI, respectively, given that these two features are the ones most frequently reported in ALS patients [1, 2, 3, 4, 15]. Moreover, patients reportedly displaying psychotic symptoms with or without other behavioral changes were addressed as a separate category given the relevance attributed to this feature by Strong's criteria as far as the definition of frontotemporal dementia (FTD) is concerned.

### 2.3 Statistics

After computing the number of patients performing defectively on each task of the cognitive section of the ECAS by applying Poletti et al.'s [16] age‐ and education‐stratified, single item‐level cutoffs, as well as that of patients showing positive symptom clusters from the ECAS‐CI, the frequency and percentage of individual MCI and MBI categories were computed. MCI and MBI categories were then classified as single‐ vs. multiple‐domain by prioritizing executive dysfunction and apathy, respectively as outlined above. Finally, the frequency and percentage of patients showing mild cognitive and behavioral impairment (MCBI) [9, 10] were computed by crossing MCI and MBI categories. A χ^2^‐test was also run to test whether MCI and MBI categories clustered in specific patterns.

Analyses were run via jamovi 2.3 (https://www.jamovi.org/).

## 3 Results

Patients' background and neuropsychological measures are reported in Table S1.

**FIGURE 1:**
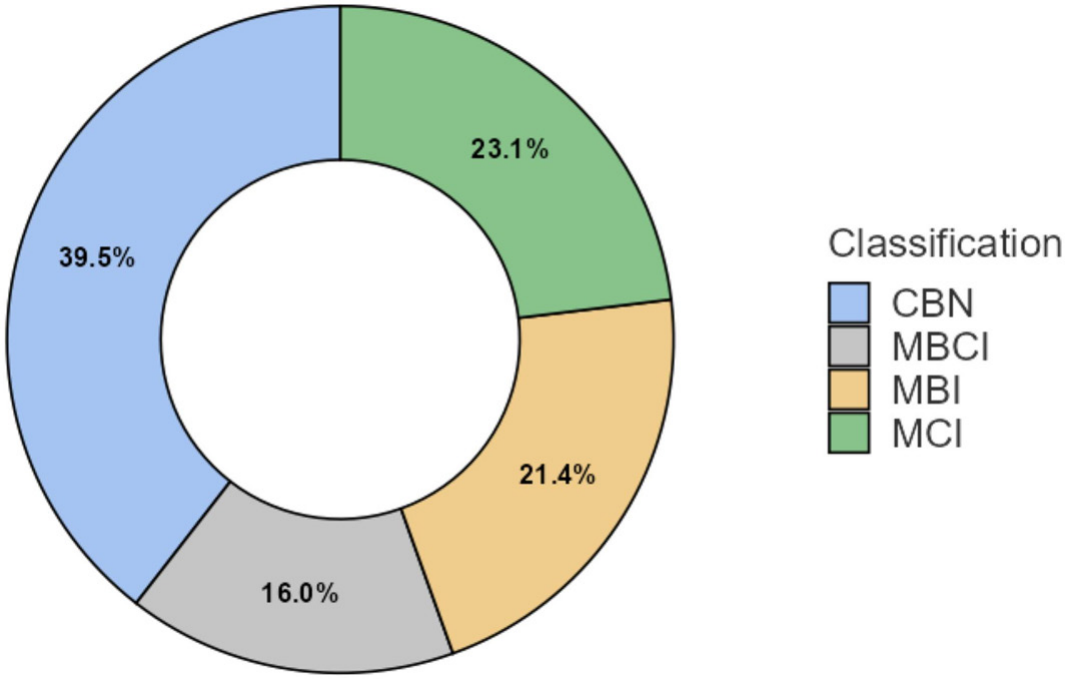
Pie chart for the distribution of the whole cohort (*N* = 901) according to the current neuropsychological classification. CBN, cognitively and behaviourally normal; MBI, mild behavioral impairment; MCBI, mild cognitive and behavioral impairment; MCI, mild cognitive impairment.

Tables 1 and 2 show the original MCI and MBI categories, respectively. MCI was detected in 352 patients (39% out of the whole cohort), whilst MBI was detected in 337 (37% of the whole cohort). Overall, 60% of patients out of the whole cohort were classified as either MCI, MBI or both (Figure 1). With regard to MCI, the vast majority of patients (63%) presented with isolated executive functioning deficits, with the second most frequent category being the co‐occurrence of executive dysfunction and language impairment (11%). As to MBI classifications, the most represented one was standalone apathy (41%), followed by the co‐occurrence of apathy and loss of sympathy/empathy (24%).

**TABLE 1:** Original MCI categories (*N* = 352, 39% of the whole cohort).

MCI	Counts	Percentages
Executive dysfunction	222	63%
Executive dysfunction + Language impairment	39	11%
Executive dysfunction + Memory impairment	25	7%
Memory impairment	19	5%
Language impairment	17	5%
Executive dysfunction + Language impairment + Memory impairment	14	4%
Visuo‐spatial impairment	7	2%
Executive dysfunction + Visuo‐spatial impairment	2	1%
Executive dysfunction + Language impairment + Memory impairment + Visuo‐spatial impairment	2	1%
Language impairment + Memory impairment	2	1%
Executive dysfunction + Language impairment + Visuo‐spatial impairment	1	< 1%
Executive dysfunction + Memory impairment + Visuo‐spatial impairment	1	< 1%
Language impairment + Visuo‐spatial impairment	1	< 1%

Abbreviation: MCI, mild cognitive impairment.

**TABLE 2:** Original MBI categories (*N* = 337, 37% of the whole cohort).

MBI	*N*	%
Apathy	135	40%
Apathy + Loss of sympathy/empathy	81	24%
Apathy + Loss of sympathy/empathy + Perseveration	20	6%
Apathy + Loss of sympathy/empathy + Perseveration + Altered eating behavior	8	2%
Apathy + Altered eating behavior	8	2%
Apathy + Perseveration	8	2%
Disinhibition + Loss of sympathy/empathy	7	2%
Apathy + Disinhibition	6	2%
Apathy + Disinhibition + Loss of sympathy/empathy + Perseveration	6	2%
Apathy + Disinhibition + Perseveration	6	2%
Apathy + Disinhibition + Loss of sympathy/empathy	5	1%
Psychosis	5	1%
Loss of sympathy/empathy + Perseveration	4	1%
Apathy + Loss of sympathy/empathy + Altered eating behavior	4	1%
Disinhibition + Perseveration	4	1%
Apathy + Perseveration + Altered eating behavior	3	1%
Disinhibition + Perseveration + Altered eating behavior	3	1%
Apathy + Disinhibition + Loss of sympathy/empathy + Perseveration + Altered eating behavior	3	1%
Perseveration + Altered eating behavior	3	1%
Loss of sympathy/empathy + Altered eating behavior	2	1%
Apathy + Psychosis	2	1%
Altered eating behavior + Psychosis	2	1%
Loss of sympathy/empathy + Perseveration + Altered eating behavior	1	< 1%
Loss of sympathy/empathy + Psychosis	1	< 1%
Apathy + Loss of sympathy/empathy + Perseveration + Psychosis	1	< 1%
Apathy + Disinhibition + Loss of sympathy/empathy + Altered eating behavior	1	< 1%
Apathy + Disinhibition + Loss of sympathy/empathy + Psychosis	1	< 1%
Apathy + Disinhibition + Altered eating behavior	1	< 1%
Apathy + Disinhibition + Perseveration + Altered eating behavior	1	< 1%
Disinhibition + Loss of sympathy/empathy + Perseveration	1	< 1%
Disinhibition + Loss of sympathy/empathy + Perseveration + Altered eating behavior	1	< 1%
Disinhibition + Altered eating behavior + Psychosis	1	< 1%
Altered eating behavior + Perseveration	1	< 1%
Apathy + Disinhibition + Loss of sympathy/empathy + Perseveration + Altered eating behavior + Psychosis	1	< 1%

Abbreviations: AEB, altered eating behavior; Apa., Apathy; Disin., disinhibition; LS/E, loss of sympathy/empathy; MBI, mild behavioral impairment; Persev., perseveration.

**FIGURE 2:**
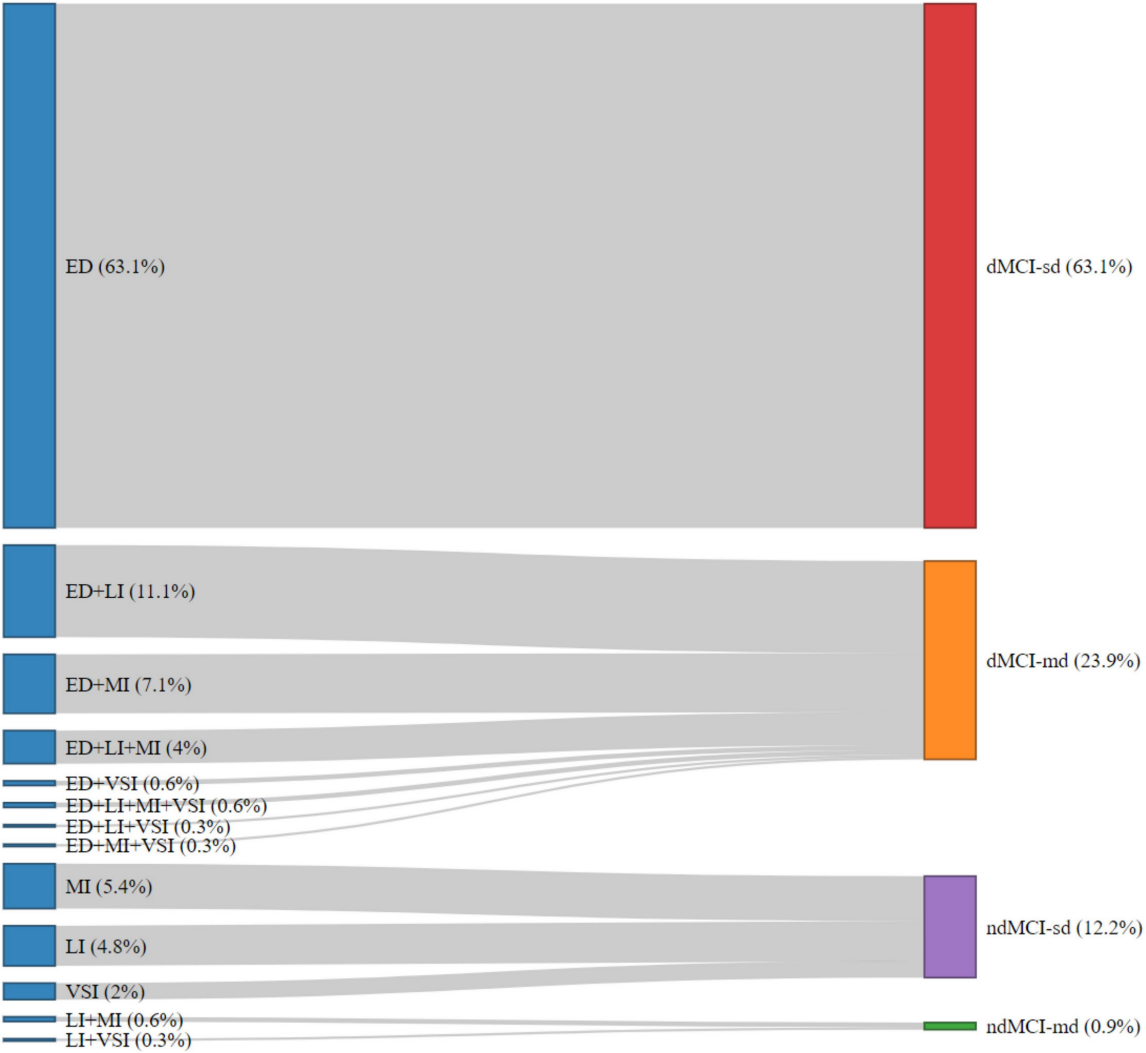
Sankey chart for the re‐classification of original MCI categories into derived MCI sub‐phenotypes. dMCI‐md, dysexecutive MCI—multiple‐domain; dMCI‐sd, dysexecutive MCI—single‐domain; ED, executive dysfunction; LI, language impairment; MCI, mild cognitive impairment; MI, memory impairment; ndMCI‐md, non‐dysexecutive MCI—multiple‐domain; ndMCI‐sd, non‐dysexecutive MCI—single‐domain; VSI, visuo‐spatial impairment.

**FIGURE 3:**
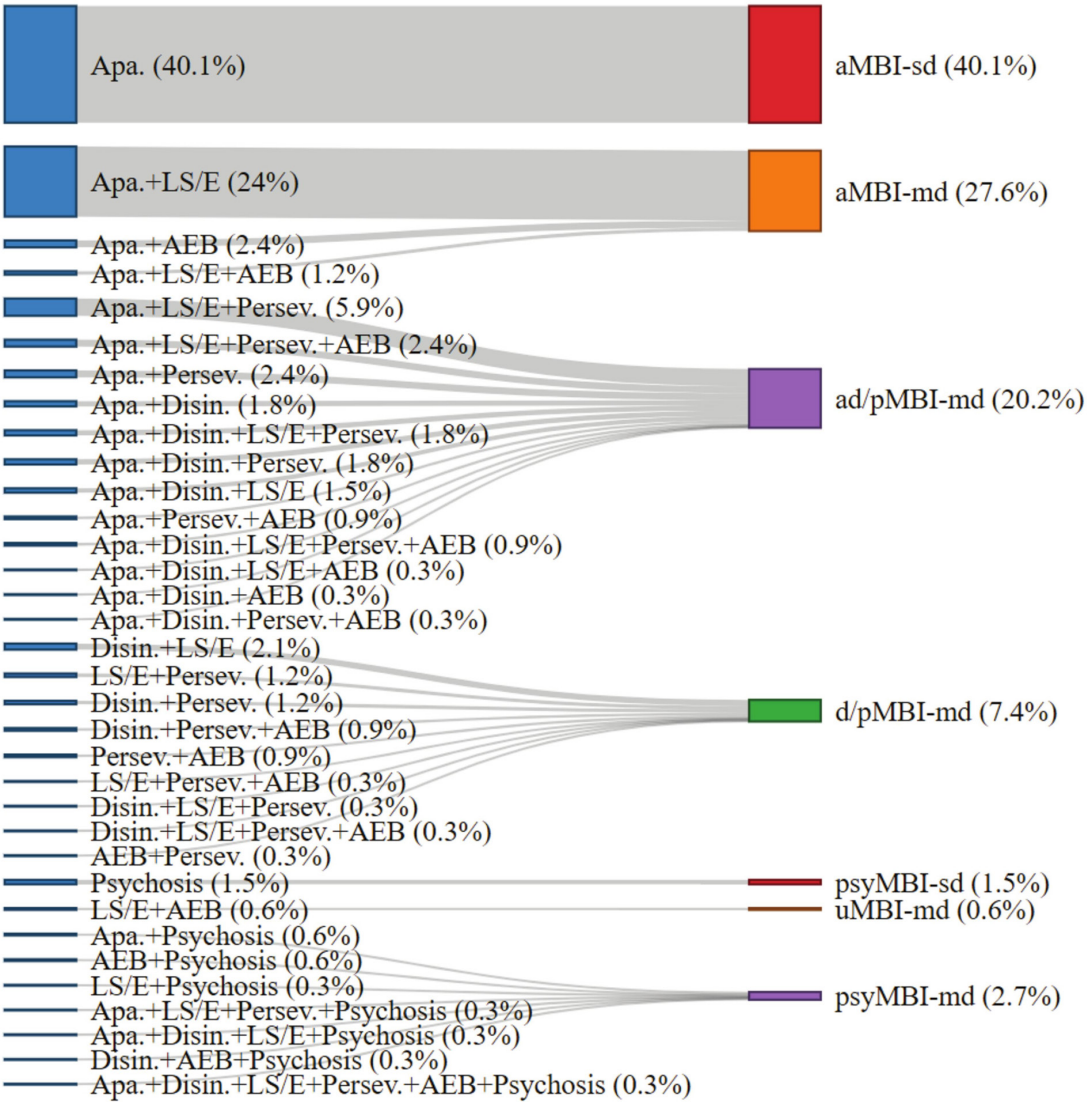
Sankey chart for the re‐classification of original MBI categories into derived MBI sub‐phenotypes. ad/pMBI‐md, apathetic‐disinihibited/perseverative MBI—multiple‐domain; AEB, altered eating behavior; aMBI‐md, apathetic MBI—multiple‐domain; aMBI‐sd, apathetic MBI—single‐domain; Apa., apathy; Disin., disinhibition; d/pMBI‐md, disinihibited/perseverative MBI—multiple‐domain; LS/E, loss of sympathy/empathy; MBI, mild behavioral impairment; MBI, mild behavioral impairment; Persev., perseveration; psyMBI‐md, psychotic MBI—multiple‐domain; psyMBI‐sd, psychotic MBI—single‐domain; uMBI‐md, unspecified MBI—multiple domain; uMBI‐md, unspecified MBI—multiple‐domain.

**FIGURE 4:**
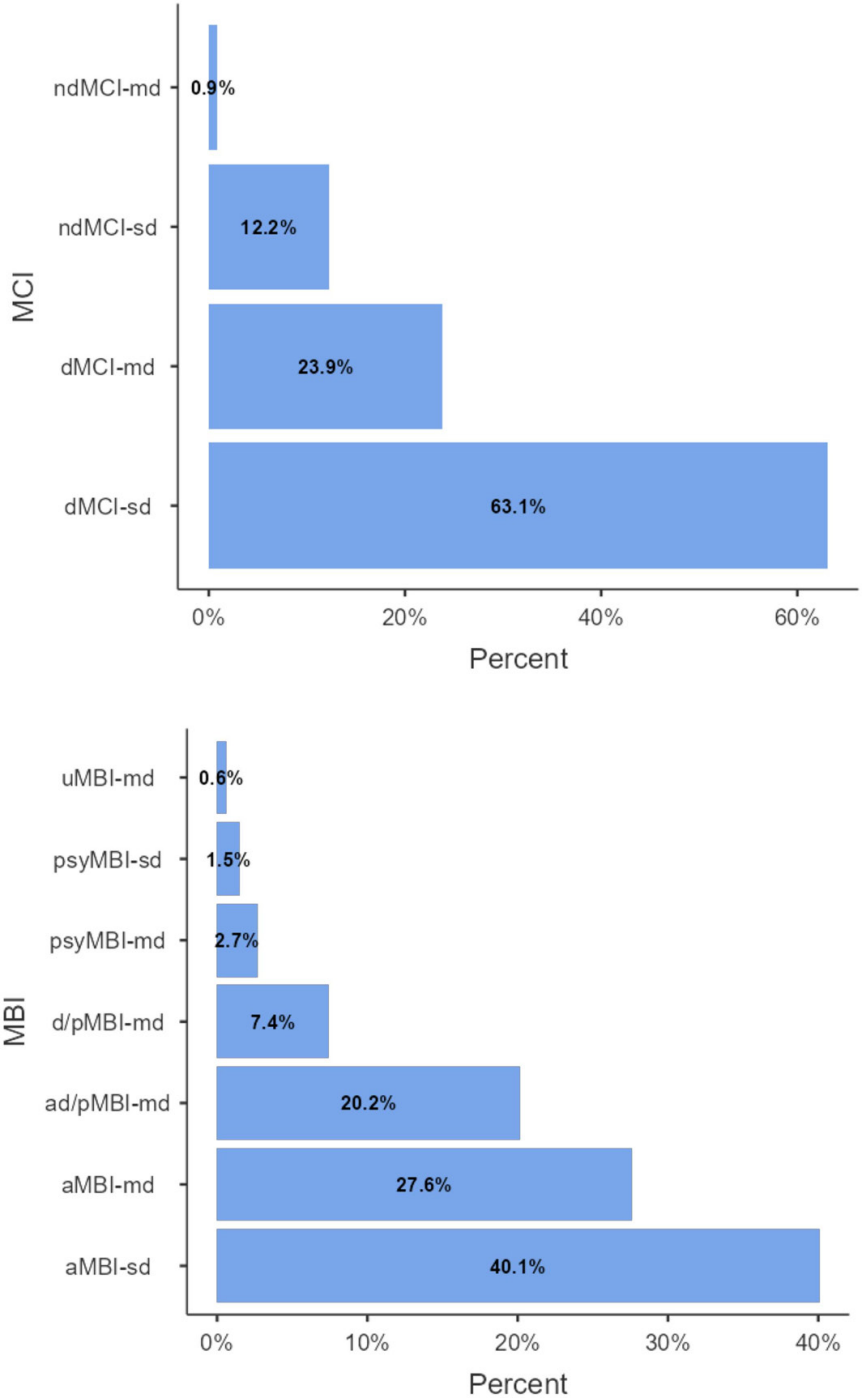
Distribution of MCI (left) and MBI (right) sub‐phenotypes. ad/pMBI‐md, apathetic‐disinihibited/perseverative MBI—multiple‐domain; aMBI‐md, apathetic MBI—multiple‐domain; aMBI‐sd, apathetic MBI—single‐domain; d/pMBI‐md, disinihibited/perseverative MBI—multiple‐domain; dMCI‐md, dysexecutive MCI—multiple‐domain; dMCI‐sd, dysexecutive MCI—single‐domain; MBI, mild behavioral impairment; MCI, mild cognitive impairment; ndMCI‐md, non‐dysexecutive MCI—multiple‐domain; ndMCI‐sd, non‐dysexecutive MCI—single‐domain; psyMBI‐md, psychotic MBI—multiple‐domain; psyMBI‐sd, psychotic MBI—single‐domain; uMBI‐md, unspecified MBI—multiple domain; uMBI‐md, unspecified MBI—multiple‐domain.

Figures 2 and 3 depict the Sankey charts for the re‐classifications of original MCI/MBI categories into the derived neuropsychological sub‐phenotypes, which are further displayed as horizontal histograms in Figure 4. The numeric values of these re‐classifications are reported in Tables S2 and S3.

Briefly, as to MCI, four categories were identified: (1) single‐domain, dysexecutive MCI (dMCI‐sd), accounting for 63% of patients with MCI—i.e., patients with isolated executive dysfunction in the absence of further cognitive deficits; (2) multiple‐domain, dysexecutive MCI (dMCI‐md), accounting for 24% of patients with MCI—i.e., patients with executive dysfunction and other cognitive deficits within the language, memory and/or visuo‐spatial domains; (3) single‐domain, non‐dysexecutive MCI (ndMCI‐sd), accounting for 12% of MCI patients—i.e., those presenting with isolated language, memory or visuospatial impairments; (4) multiple‐domain, non‐dysexecutive MCI (ndMCI‐sd), accounting for about 1% of MCI patients—i.e., those presenting with a combination of at least two deficits within the language, memory or visuospatial domains. Table 3 reports the proposed criteria for MCI in ALS according to these four categories.

**TABLE 3:** Operationalization of MCI sub‐phenotypes in ALS.

Sub‐phenotype	Operationalization
Dysexecutive MCI—single domain (dMCI‐sd)	At least one of the following criteria is met (A.1‐A.2): A.1: ≥ 1 below‐cutoff subtest from the ECAS‐Fluency^a^ (i.e., *Verbal fluency—S* and *Verbal fluency—C*) A.2: ≥ 2 below‐cutoff subtests from the ECAS‐Executive^a^ (i.e., *Backward digit span*, *Alternation*, *Sentence completion*, and *Social cognition*) AND All of the following criteria are met (A.3‐A.5): A.3: ≥ 2 above‐cutoff subtests from the ECAS‐Language^a^ (i.e., *Naming*, *Comprehension* and *Spelling*) A.4: ≥ 2 above‐cutoff subtests from the ECAS‐Memory^a^ (i.e., *Immediate recall*, *Retention score* and *Recognition*) A.5: ≥ 2 above‐cutoff subtests from the ECAS‐Visuospatial^a^ (i.e., *Number position*, *Dot counting* and *Cube counting*)
Dysexecutive MCI—multiple domain (dMCI‐md)	Either criterion A.1 or criterion A.2 is met AND At least one of the following criteria is met (B.1‐B.3) B.1: ≥ 2 below‐cutoff subtests from the ECAS‐Language^a^ (i.e., *Naming*, *Comprehension* and *Spelling*) B.2: ≥ 2 below‐cutoff subtests from the ECAS‐Memory^a^ (i.e., *Immediate recall*, *Retention score* and *Recognition*) B.3: ≥ 2 below‐cutoff subtests from the ECAS‐Visuospatial^a^ (i.e., *Number position*, *Dot counting* and *Cube counting*)
Non‐dysexecutive MCI—single domain (ndMCI‐sd)	Neither criterion A.1 nor criterion A.2 are met AND Only one of the following criteria is met (C.1‐C.3): C.1: ≥ 2 below‐cutoff subtests from the ECAS‐Language^a^ (i.e., *Naming*, *Comprehension* and *Spelling*) C.2: ≥ 2 below‐cutoff subtests from the ECAS‐Memory^a^ (i.e., *Immediate recall*, *Retention score* and *Recognition*) C.3: ≥ 2 below‐cutoff subtests from the ECAS‐Visuospatial^a^ (i.e., *Number position*, *Dot counting* and *Cube counting*)
Non‐dysexecutive MCI—multiple domain (ndMCI‐md)	Neither criterion A.1 nor criterion A.2 are met AND At least two criteria among C.1, C.2 and C.3 are met

Abbreviations: ALS, amyotrophic laterals sclerosis; ECAS, Edinburgh Cognitive and Behavioral ALS Screen; MCI, mild cognitive impairment.

^a^ single subtest‐level cutoffs according to Poletti et al. [16].

As to MBI, the following classifications were derived: (1) single‐domain, apathetic MBI (aMBI‐sd), accounting for 40% of MBI patients—namely, those with isolated apathy without further behavioral changes; (2) multiple‐domain, apathetic MBI (aMBI‐md), accounting for 28% of MBI patients—i.e., those with apathy and at least one behavioral change among loss of sympathy/empathy or altered eating behaviors; (3) multiple‐domain, apathetic and disinhibited/perseverative MBI (ad/pMBI‐MD), accounting for 20% of patients with MBI—i.e., those showing apathy and at least one positive cluster between disinhibition and perseveration, with or without further behavioral changes (excluding psychosis), (4) multiple‐domain, disinhibited/perseverative MBI (ad/pMBI‐md), accounting for 7% of patients—i.e., those with at least one positive cluster among disinhibition and perseveration and at least an additional behavioral change excluding apathy and psychotic symptoms, (5) single‐domain, psychotic MBI (psyMBI‐sd), accounting for 2% of patients—i.e., those with psychotic symptoms in the absence of further behavioral alterations and (6) multiple‐domain, psychotic MBI (psyMBI‐md), accounting for 3% of patients—i.e., those with psychotic symptoms and at least another positive cluster among apathy, loss of sympathy/empathy, disinhibition, perseveration and altered eating behaviors. A seventh, unclassifiable category (uMBI‐md) was drafted for those patients (1%) that could not fall under the previous four MBI categories. Table 4 reports the proposed criteria for MBI in ALS according to these four categories.

The prevalence of MCBI was 16% (144 patients out of the whole cohort). No association was found between specific MCI and MBI sub‐phenotypes (χ^2^(18) = 22.78; *p* = 0.199). Figure S1 displays the bar chart for the crossing of MCI and MBI categories.

**TABLE 4:** Operationalization of MBI sub‐phenotypes in ALS.

Sub‐phenotype	Operationalization
Apathetic MBI—single domain (aMBI‐sd)	A.1: a positive *Apathy* ^*a*^ item from the ECAS‐CI AND A.2: no positive symptom clusters from the ECAS‐CI other than *Apathy* (i.e., *Disinhibition* ^b^, *Loss of sympathy/empathy* ^c^, *Perseveration* ^c^, *Altered eating behaviour* ^c^ and *Psychosis* ^b^)
Apathetic MBI—multiple domain (aMBI‐smd)	Criterion A.1 is met AND All of the following criteria are met (B.1‐B.3): B.1: ≥ 1 positive symptom clusters among *Loss of sympathy/empathy* ^c^ and *Altered eating behaviour* ^c^ B.2: no positive symptom clusters among *Disinhibition* ^b^ and *Perseveration* ^c^ B.3: no positive items within the *Psychosis* ^b^ cluster
Apathetic‐disinhibited/perserverative MBI—multiple domain (ad/pMCI‐md)	Criteria A.1 and B.3 are met AND C: ≥ 1 positive symptom clusters among *Disinhibition* ^b^ and *Perseveration* ^c^, with or without positive symptom clusters among *Loss of sympathy/empathy* ^c^ and *Altered eating behaviour* ^c^
Disinhibited/perserverative MBI—multiple domain (d/pMCI‐md)	Criterion A.1 is not met AND Criterion B.3 is met AND D: ≥ 1 positive symptom clusters among *Disinhibition* ^b^ and *Perseveration* ^c^, together with ≥ 1 positive symptom clusters among *Loss of sympathy/empathy* ^c^ and *Altered eating behaviour* ^c^
Psychotic MBI—single domain (psyMBI‐sd)	E.1: ≥ 1 positive item from the *Psychosis* ^b^ cluster from the ECAS‐CI AND Criterion A.1 is not met E.2: no positive symptom clusters from the ECAS‐CI among *Disinhibition* ^b^, *Loss of sympathy/empathy* ^c^, *Perseveration* ^c^ and *Altered eating behaviour* ^c^
Psychotic MBI—multiple domain (psyMBI‐md)	Criterion E.1 is met AND At least one the following criteria is met (B.1‐B.3): A.1 F: ≥ 1 positive symptom clusters from the ECAS‐CI among *Loss of sympathy/empathy* ^c^, *Perseveration* ^c^ and *Altered eating behaviour* ^c^
Unclassifiable MBI—multiple domain (uMCI‐md)	Criteria A.1, C and D are not met AND G: ≥ 1 positive symptom clusters among *Loss of sympathy/empathy* ^c^ and *Altered eating behaviour* ^c^

Abbreviations: ALS, amyotrophic lateral sclerosis; ECAS, Edinburgh Cognitive and Behavioral ALS Screen; MBI, mild behavioral impairment.

^a^ this symptom cluster includes 1 item.

^b^ this symptom cluster includes 3 items.

^c^ this symptom cluster includes 2 items.

## 4 Discussion

The present study delivers a proposal for the neuropsychological sub‐phenotyping of non‐demented ALS patients according to an approach relying on the ECAS, as well as in pursuance of MCI and MBI frameworks adopted for other dementing conditions [7, 8, 9].

Data herewith reported might be useful for a detailed stratification of ALS patients' neuropsychological status that goes beyond Strong's criteria, which categorize individuals only on the presence or absence of cognitive and/or behavioral dysfunction without addressing the specific characteristics of these impairments. In that light, the current proposal complements Strong's criteria by addressing cognitive domains that are not encompassed within such a nosographic system—namely, memory and visuospatial ones. Importantly, our proposed MBI algorithm addresses psychotic features which, in Strong's criteria, are taken into account solely for the diagnosis of concurrent FTD. While our proposed framework will be particularly helpful in the context of furthering our understanding of the breadth of deficits within the syndromes of frontotemporal dysfunction in ALS, future studies will need to assess whether these subcategorizations have specific impact on ALS progression and survivorship—as suggested by Spisto et al.'s [7] recent work.

Interestingly, the present classifications yielded prevalence estimates for MCI, MBI and MCBI categories that are overall consistent both with a recent, large‐scaled study on frontotemporal‐*spectrum* disorders in ALS that employed Strong's criteria [15]—where the cumulative prevalence of ALSci, ALSbi and ALScbi cases was ~53% –, and with Spisto et al.’ s [7] work embracing the MBNI approach—where, excluding patients with dementia, the cumulative prevalence of MCI, MBI and MCBI cases was ~65%. Moreover, the current framework appears to align with the Miami Framework that has been recently proposed by Benatar et al. [17], which likewise identified MCI, MBI and MCBI categories.

As far as ALS patients' neuropsychological profile, this study aligns with the literature on neuropsychological changes in this population, showing that MCI profiles are dominated by dysexecutive features and language deficits and that MBI ones heavily rely on apathetic and disinhibited/perseverative features—these being the core clinical features of ALS‐related frontotemporal‐*spectrum* disorders [1, 2, 3, 4, 5, 15].

The main limitation of the present study lies in the adoption of a first‐level neuropsychological screener, i.e., the ECAS, instead of second‐level cognitive measures and in‐depth behavioral questionnaires. However, this has been justified by the fact that the vast majority of available second‐level cognitive measures, with only a few exceptions, insufficiently compensate for patients' verbal‐motor disabilities. With that being said, as long as future studies develop ALS‐adapted, second‐level cognitive measures, it is imperative for further investigations to verify the validity of the current classifications via more detailed neuropsychological measures.

A further, noteworthy limitation affecting the current investigation lies in the fact that it solely relies on psychometric measures, whilst not on patients' history and information on the potential influence of their neuropsychological status on functional independence. Hence, whilst none of these patients received a formal diagnosis of dementia, it cannot be fully ruled out that all of these patients actually presented with “mild” cognitive and/or behavioral dysfunctions. However, it should be noted that this issue, at least when referred to changes in behavior, partially reflects the scoring methods of currently available ALS‐specific instruments. In fact, available caregiver‐report semi‐structured interviews/questionnaires—such as the ECAS‐CI itself—embrace a dichotomic classification of behavioral changes, by at variance neglecting their frequency and severity. A Frequency*Severity scoring algorithm, such as the one employed within the Neuropsychiatric Inventory [18], would nevertheless be of interest when it comes to assessing ALS patients' behavioral status, as possibly informing on the actual impact of behavioral dysfunctions in ecological scenarios. It would be thus advisable for future investigations on the topic to develop ALS‐specific behavioral instruments that rate their behavioral changes beyond their mere absence or presence. Furthermore, future studies comparing ALS with non‐neurological terminal conditions are warranted to better disentangle reactive psychological distress from disease‐specific behavioral changes.

In conclusion, the present study delivers a provisional, ECAS‐based classification for the detailed neuropsychological sub‐phenotyping of non‐demented ALS patients, which, with further validation, might be useful for both research and clinical purposes.

## Author Contributions

**Monica Consonni:** conceptualization, data curation, resources, writing – original draft, writing – review and editing. **Edoardo Nicolò Aiello:** conceptualization, formal analysis, writing – original draft, writing – review and editing. **Veronica Faltracco:** data curation, writing – review and editing. **Alessandra Telesca:** data curation, writing – review and editing. **Beatrice Curti:** formal analysis, writing – original draft, writing – review and editing. **Barbara Poletti:** conceptualization, writing – original draft, writing – review and editing, resources, data curation. **Silvia Torre:** data curation, writing – review and editing. **Barbara Iazzolino:** data curation, resources, writing – original draft, writing – review and editing. **Nilo Riva:** data curation, writing – review and editing. **Giulia De Luca:** formal analysis, writing – review and editing, writing – original draft. **Stefano Messina:** data curation, writing – review and editing. **Francesca Palumbo:** data curation, writing – review and editing. **Enrica Bersano:** data curation, writing – review and editing. **Eleonora Dalla Bella:** data curation, writing – review and editing. **Francesca Frisco:** formal analysis, writing – review and editing, writing – original draft. **Michael J. Strong:** writing – review and editing. **Giuseppe Lauria:** funding acquisition, resources, writing – review and editing. **Federico Verde:** data curation, writing – review and editing. **Alessio Maranzano:** data curation, writing – review and editing. **Claudia Morelli:** data curation, writing – review and editing. **Stefano Francesco Cappa:** writing – review and editing. **Adriano Chiò:** funding acquisition, resources, writing – review and editing. **Andrea Calvo:** resources, writing – review and editing, funding acquisition. **Nicola Ticozzi:** funding acquisition, resources, writing – review and editing. **Vincenzo Silani:** funding acquisition, resources, writing – review and editing. **Arianna Moreschi:** formal analysis, writing – review and editing, writing – original draft. **Alberto Doretti:** data curation, writing – review and editing.

## Funding

This work was supported by the Italian Ministry of Health, by Fondazione Regionale per la Ricerca Biomedica, Regione Lombardia (TRANS‐ALS; grant number: 2015‐0023), by Fondo Europeo di Sviluppo Regionale, Regione Lombardia (POR FESR 2014–2020; grant number: 1157625), by Ricerca Sanitaria Finalizzata—Ministero della Salute (grant: RF‐2016‐02362405), by “Progetti di Rilevante Interesse Nazionale” programme of the Ministry of Education, University and Research (grant: 2017SNW5MB), and by Horizon 2020 (grant: RF H2020‐SC1‐DTH2020‐1, grant agreement ID: 101017598). This study was also performed under the Department of Excellence grant of the Italian Ministry of Education, University and Research to the “Rita Levi Montalcini” Department of Neuroscience, University of Torino, Italy.

## Ethics Statement

Participants provided informed consent and data were treated according to current regulations. This study was approved by the Ethics Committee of IRCCS Istituto Auxologico Italiano (ID: 2013_06_25), by the Ethics Committee of Fondazione IRCCS Istituto Neurologico Carlo Besta (ID: 71/2015; 2017/06/07, Excerpt 6/41; 2020/06/17, Excerpt 01/73; 2023/01/18, Excerpt 06/11), and by the Ethics Committee of the ALS Expert Center of Torino, Azienda Ospedaliero Universitaria Città della Salute e della Scienza (ID: #0038876).

## Conflicts of Interest

V.S.received compensation for consulting services and/or speaking activities from AveXis, Cytokinetics, Italfarmaco, Liquidweb S.r.l., and Novartis Pharma AG, receives or has received research supports from the Italian Ministry of Health, AriSLA, and E‐Rare Joint Transnational Call. He is in the Editorial Board of *Amyotrophic Lateral Sclerosis and Frontotemporal Degeneration*, *European Neurology*, *American Journal of Neurodegenerative Diseases*, *Frontiers in Neurology*. B.P. received compensation for consulting services and/or speaking activities from Liquidweb S.r.l. B.P. is Associate Editor for *Frontier in Neuroscience*. N.T.received compensation for consulting services and/or speaking fees from Amylyx Pharmaceuticals, Biogen, Italfarmaco and Zambon Biotech SA. He is Associate Editor for *Frontiers in Aging Neuroscience*. F.V. is Associated Editor for *Journal of Alzheimer's Disease*. E.N.A. serves as an Editorial Board Member for *BMC Neurology*. A.Ch. is a member of the following Scientific Advisory Boards: Mitsubishi Tanabe, Roche, Biogen, Denali Pharma, Cytokinetics, Amylyx Pharmaceuticals, VectorY, Ferrer and Zambon Biotech; he received a scientific grant from Biogen; he is a member of the following Drug Safety and Monitoring Boards: AB Science, Verge, Corcept and Eli Lilly; A. Ca. has received a research grant from Cytokinetics. The other Authors have nothing to declare.

## Supporting information


**Figure S1:** Bar charts for the crossing between MCI and MBI categories (*N* = 144 MCBI patients, 16%).
Notes. MCI = mild cognitive impairment; MBI = mild behavioral impairment; MCBI = mild cognitive and behavioral impairment; dMCI‐sd = dysexecutive MCI—single‐domain; dMCI‐md = dysexecutive MCI—multiple‐domain; ndMCI‐sd = non‐dysexecutive MCI—single‐domain; ndMCI‐md = non‐dysexecutive MCI—multiple‐domain; aMBI‐sd = apathetic MBI—single‐domain; aMBI‐md = apathetic MBI—multiple‐domain; ad/pMBI‐md = apathetic‐disinihibited/perseverative MBI—multiple‐domain; d/pMBI‐md = disinihibited/perseverative MBI—multiple‐domain; uMBI‐md = unspecified MBI—multiple‐domain; psyMBI‐sd = psychotic MBI—single‐domain; psyMBI‐md = psychotic MBI—multiple‐domain; uMBI‐md = unspecified MBI—multiple domain. Within each bar, the stacks display how many MCI patients were concurrently classified as having MBI—with MCI sub‐phenotypes being distributed along the *x*‐axis and MBI ones being represented by different colors labeled in the legend on the right.

**Table S1:** Patients' background and clinical measures.
Notes. ALSFRS‐*R* = Amyotrophic Lateral Sclerosis Functional Rating Scale‐Revised; ΔFS = progression rate; ECAS = Edinburgh Cognitive and Behavioral ALS Screen; MiToS = Milano‐Torino staging system; ECAS‐CI = ECAS Carer Interview; ALS = amyotrophic lateral sclerosis; cbn = cognitively and behaviourally normal; ci = cognitively impaired; bi = behaviourally impaired; cbi = cognitively and behaviourally impaired. ^a^data available for *N* = 854 patients; ^b^data available for *N* = 827 patients; ^c^data available for *N* = 788 patient; ^d^data available for *N* = 720 patients.

**Table S2:** Numerical values for the re‐classification of original MCI categories into MCI sub‐phenotypes.
Notes. MCI = mild cognitive impairment; ED = executive dysfunction; LI = language impairment; MI = memory impairment; VSI = visuo‐spatial impairment; dMCI‐sd = dysexecutive MCI—single‐domain; dMCI‐md = dysexecutive MCI—multiple‐domain; ndMCI‐sd = non‐dysexecutive MCI—single‐domain; ndMCI‐md = non‐dysexecutive MCI—multiple‐domain.

**Table S3:** Numerical values for the re‐classification of original MBI categories into MBI sub‐phenotypes.
Notes. MBI = mild behavioral impairment; Apa. = apathy; Disin. = disinhibition; Persev. = perseveration; AEB = altered eating behavior; LS/E = loss of sympathy/empathy; MBI = mild behavioral impairment; aMBI‐sd = apathetic MBI—single‐domain; aMBI‐md = apathetic MBI—multiple‐domain; ad/pMBI‐md = apathetic‐disinihibited/perseverative MBI—multiple‐domain; d/pMBI‐md = disinihibited/perseverative MBI—multiple‐domain; uMBI‐md = unspecified MBI—multiple‐domain; psyMBI‐sd = psychotic MBI—single‐domain; psyMBI‐md = psychotic MBI—multiple‐domain; uMBI‐md = unspecified MBI—multiple domain.

## Data Availability

Datasets associated with the present study cannot be made publicly available on ethical‐legal grounds, but raw data from the Corresponding Author's primary Institution have been stored on an online repository with restricted access (https://doi.org/10.5281/zenodo.21363009) and can be made available upon reasonable request of interested researchers to the Corresponding Author(s), who will forward a request for a data transfer agreement to the relevant Ethical Committee(s).
