# Supplementary material for: Neuropsychological Sub‐Phenotypes in Amyotrophic Lateral Sclerosis
Source: Eur J Neurol. 2026 Aug 3;33(8):e70706. doi: 10.1111/ene.70706 (PMC13431129; doi:10.1111/ene.70706)
Supplement: Supplementary file 1 — Figure S1: Bar charts for the crossing between MCI and MBI categories (N = 144 MCBI patients, 16%). Notes. MCI = mild cognitive impairment; MBI = mild behavioral impairment; MCBI = mild cognitive and behavioral impairment; dMCI‐sd = dysexecutive MCI—single‐domain; dMCI‐md = dysexecutive MCI—multiple‐domain; ndMCI‐sd = non‐dysexecutive MCI—single‐domain; ndMCI‐md = non‐dysexecutive MCI—multiple‐domain; aMBI‐sd = apathetic MBI—single‐domain; aMBI‐md = apathetic MBI—multiple‐domain; ad/pMBI‐md = apathetic‐disinihibited/perseverative MBI—multiple‐domain; d/pMBI‐md = disinihibited/perseverative MBI—multiple‐domain; uMBI‐md = unspecified MBI—multiple‐domain; psyMBI‐sd = psychotic MBI—single‐domain; psyMBI‐md = psychotic MBI—multiple‐domain; uMBI‐md = unspecified MBI—multiple domain. Within each bar, the stacks display how many MCI patients were concurrently classified as having MBI—with MCI sub‐phenotypes being distributed along the x‐axis and MBI ones being represented by different colors labeled in the legend on the right. [file ENE-33-e70706-s002.docx]

**Supplementary figure 1.** Bar charts for the crossing between MCI and MBI categories (N=144 MCBI patients, 16%).


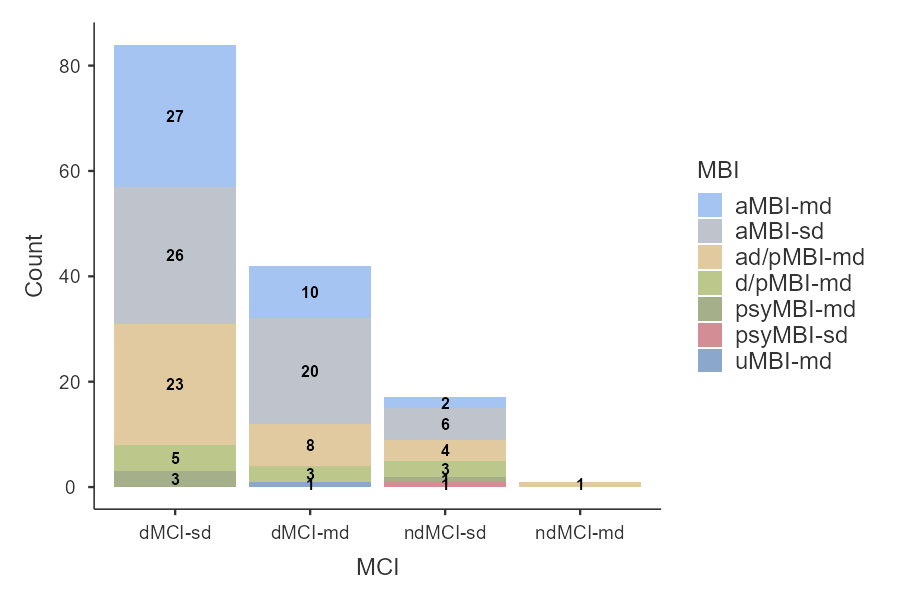


**Notes.** MCI=mild cognitive impairment; MBI=mild behavioural impairment; MCBI=mild cognitive and behavioural impairment; dMCI-sd=dysexecutive MCI – single-domain; dMCI-md=dysexecutive MCI – multiple-domain; ndMCI-sd=non-dysexecutive MCI – single-domain; ndMCI-md=non-dysexecutive MCI – multiple-domain; aMBI-sd=apathetic MBI – single-domain; aMBI-md=apathetic MBI – multiple-domain; ad/pMBI-md=apathetic-disinihibited/perseverative MBI – multiple-domain; d/pMBI-md=disinihibited/perseverative MBI – multiple-domain; uMBI-md=unspecified MBI – multiple-domain; psyMBI-sd=psychotic MBI – single-domain; psyMBI-md=psychotic MBI – multiple-domain; uMBI-md=unspecified MBI – multiple domain. Within each bar, the stacks display how many MCI patients were concurrently classified as having MBI – with MCI sub-phenotypes being distributed along the *x*-axis and MBI ones being represented by different colors labeled in the legend on the right.
