# Supplementary material for: Neuropsychological Sub‐Phenotypes in Amyotrophic Lateral Sclerosis
Source: Eur J Neurol. 2026 Aug 3;33(8):e70706. doi: 10.1111/ene.70706 (PMC13431129; doi:10.1111/ene.70706)
Supplement: Supplementary file 2 — Table S1: Patients' background and clinical measures. Notes. ALSFRS‐R = Amyotrophic Lateral Sclerosis Functional Rating Scale‐Revised; ΔFS = progression rate; ECAS = Edinburgh Cognitive and Behavioral ALS Screen; MiToS = Milano‐Torino staging system; ECAS‐CI = ECAS Carer Interview; ALS = amyotrophic lateral sclerosis; cbn = cognitively and behaviourally normal; ci = cognitively impaired; bi = behaviourally impaired; cbi = cognitively and behaviourally impaired. adata available for N = 854 patients; bdata available for N = 827 patients; cdata available for N = 788 patient; ddata available for N = 720 patients. [file ENE-33-e70706-s001.docx]

**Supplementary Table 1.** Patients’ background and clinical measures

|  | **Total** |
| --- | --- |
| ***N*** | 901 |
| **Age (years)** | 63.5 ± 10.99 (20-88) |
| **Sex (male/female)** | 56%/44% |
| **Education (years)** | 11.39 ± 4.27 (3-25) |
| **Disease duration (months)^a^** | 19.25 ± 19.27 (1-264) |
| **ALSFRS-R^b^** | 38.77 ± 5.85 (12-47) |
| **ΔFS^c^** | .83 ± .89 (.03-7) |
| **King’s (Stage 1/2/3/4 %)^b^** | 38%/33%/25%/4% |
| **MiToS (Stage 0/1/2/3 %)^b^** | 78%/20%/2%/0% |
| **Genetics (*N*)^d^** |  |
| *C9orf72* | 56 |
| *SOD1* | 25 |
| *TARDBP* | 21 |
| *FUS* | 3 |
| *OPTN* | 4 |
| *SQSTM1* | 3 |
| Other | 17 |
| **ECAS [impaired %]** |  |
| **Total** | 98.86 ± 19.05 (24-133) [33%] |
| **ALS-specific** | 72.41 ± 15.69 (13-98) [33%] |
| **ALS-nonspecific** | 26.45 ± 4.95 (6-36) [19%] |
| **Language** | 23.55 ± 3.95 (1-28) [20%] |
| Naming | 6.73 ± 1.4 (0-8) [15%] |
| Comprehension | 7.63 ± 0.74 (0-8) [9%] |
| Spelling | 9.19 ± 2.87 (0-12) [16%] |
| **Fluency** | 16.49 ± 5.74 (0-24) [20%] |
| Verbal fluency - S | 8.48 ± 3.01 (0-12) [20%] |
| Verba fluency - C | 8.02 ± 3.45 (0-12) [19%] |
| **Executive** | 32.36 ± 8.5 (6-48) [31%] |
| Backward digit span | 5.26 ± 1.89 (0-12) [1%] |
| Alternation | 8.63 ± 4.28 (0-12) [32%] |
| Sentence completion | 8.9 ± 2.63 (0-12) [25%] |
| Social cognition | 9.57 ± 3.23 (0-12) [11%] |
| **Memory** | 15.17 ± 4.41 (0-24) [16%] |
| Immediate recall | 5.12 ± 1.98 (0-10) [7%] |
| Retention score | 7.83 ± 2.66 (0-10) [20%] |
| Recognition | 2.22 ± 1.23 (0-4) [12%] |
| **Visuospatial** | 11.28 ± 1.2 (4-12) [9%] |
| Number position | 3.82 ± 0.57 (0-4) [12%] |
| Dot-counting | 3.87 ± 0.39 (1-4) [1%] |
| Cube-counting | 3.59 ± 0.8 (0-4) [5%] |
| **ECAS-CI** |  |
| **Total** | 0.96 ± 1.48 (0-10) |
| **Disinhibition** | 7% |
| Socially inappropriate behaviour | 3% |
| Loss of manners/decorum | 3% |
| Impulsiveness | 3% |
| **Loss of sympathy/empathy** | 19% |
| Diminished response to others’ needs/feelings | 9% |
| Diminished social interest | 15% |
| **Apathy** | 33% |
| **Perseveration** | 11% |
| Simple repetitive movements | 9% |
| Complex/compulsive/ritualistic behaviour | 9% |
| **Altered eating behaviour** | 7% |
| Altered food preferences | 5% |
| Binge eating/hyperorality | 5% |
| **Psychosis** | 2% |
| Suspicion | 1% |
| Delusions | <1% |
| Hallucinations | <1% |

**Notes.** ALSFRS-R=Amyotrophic Lateral Sclerosis Functional Rating Scale-Revised; ΔFS=progression rate; ECAS=Edinburgh Cognitive and Behavioural ALS Screen; MiToS=Milano-Torino staging system; ECAS-CI=ECAS Carer Interview; ALS=amyotrophic lateral sclerosis; cbn=cognitively and behaviourally normal; ci=cognitively impaired; bi=behaviourally impaired; cbi=cognitively and behaviourally impaired. ^a^data available for *N*=854 patients; ^b^data available for *N*=827 patients; ^c^data available for *N*=788 patient; ^d^data available for *N*=720 patients.
