# Supplementary material for: Neuropsychological Sub‐Phenotypes in Amyotrophic Lateral Sclerosis
Source: Eur J Neurol. 2026 Aug 3;33(8):e70706. doi: 10.1111/ene.70706 (PMC13431129; doi:10.1111/ene.70706)
Supplement: Supplementary file 3 — Table S2: Numerical values for the re‐classification of original MCI categories into MCI sub‐phenotypes. Notes. MCI = mild cognitive impairment; ED = executive dysfunction; LI = language impairment; MI = memory impairment; VSI = visuo‐spatial impairment; dMCI‐sd = dysexecutive MCI—single‐domain; dMCI‐md = dysexecutive MCI—multiple‐domain; ndMCI‐sd = non‐dysexecutive MCI—single‐domain; ndMCI‐md = non‐dysexecutive MCI—multiple‐domain. [file ENE-33-e70706-s003.docx]

**Supplementary Table 2.** Numerical values for the re-classification of original MCI categories into MCI sub-phenotypes.

| **Original MCI category** | **MCI sub-phenotype** | **Frequency** |
| --- | --- | --- |
| ED | dMCI-sd | 222 |
| ED+LI | dMCI-md | 39 |
| ED+MI | dMCI-md | 25 |
| MI | ndMCI-sd | 19 |
| LI | ndMCI-sd | 17 |
| ED+LI+MI | dMCI-md | 14 |
| VSI | ndMCI-sd | 7 |
| ED+VSI | dMCI-md | 2 |
| ED+LI+MI+VSI | dMCI-md | 2 |
| LI+MI | ndMCI-md | 2 |
| ED+LI+VSI | dMCI-md | 1 |
| ED+MI+VSI | dMCI-md | 1 |
| LI+VSI | ndMCI-md | 1 |

**Notes.** MCI=mild cognitive impairment; ED=executive dysfunction; LI=language impairment; MI=memory impairment; VSI=visuo-spatial impairment; dMCI-sd=dysexecutive MCI – single-domain; dMCI-md=dysexecutive MCI – multiple-domain; ndMCI-sd=non-dysexecutive MCI – single-domain; ndMCI-md=non-dysexecutive MCI – multiple-domain.
