# Supplementary material for: Neuropsychological Sub‐Phenotypes in Amyotrophic Lateral Sclerosis
Source: Eur J Neurol. 2026 Aug 3;33(8):e70706. doi: 10.1111/ene.70706 (PMC13431129; doi:10.1111/ene.70706)
Supplement: Supplementary file 4 — Table S3: Numerical values for the re‐classification of original MBI categories into MBI sub‐phenotypes. Notes. MBI = mild behavioral impairment; Apa. = apathy; Disin. = disinhibition; Persev. = perseveration; AEB = altered eating behavior; LS/E = loss of sympathy/empathy; MBI = mild behavioral impairment; aMBI‐sd = apathetic MBI—single‐domain; aMBI‐md = apathetic MBI—multiple‐domain; ad/pMBI‐md = apathetic‐disinihibited/perseverative MBI—multiple‐domain; d/pMBI‐md = disinihibited/perseverative MBI—multiple‐domain; uMBI‐md = unspecified MBI—multiple‐domain; psyMBI‐sd = psychotic MBI—single‐domain; psyMBI‐md = psychotic MBI—multiple‐domain; uMBI‐md = unspecified MBI—multiple domain. [file ENE-33-e70706-s004.docx]

**Supplementary Table 3.** Numerical values for the re-classification of original MBI categories into MBI sub-phenotypes.

| **Original MBI category** | **MBI sub-phenotype** | **Frequency** |
| --- | --- | --- |
| Apa. | aMBI-sd | 135 |
| Apa.+LS/E | aMBI-md | 81 |
| Apa.+LS/E+Persev. | ad/pMBI-md | 20 |
| Apa.+LS/E+Persev.+AEB | ad/pMBI-md | 8 |
| Apa.+AEB | aMBI-md | 8 |
| Apa.+Persev. | ad/pMBI-md | 8 |
| Disin.+LS/E | d/pMBI-md | 7 |
| Apa.+Disin. | ad/pMBI-md | 6 |
| Apa.+Disin.+LS/E+Persev. | ad/pMBI-md | 6 |
| Apa.+Disin.+Persev. | ad/pMBI-md | 6 |
| Apa.+Disin.+LS/E | ad/pMBI-md | 5 |
| Psychosis | psyMBI-sd | 5 |
| LS/E+Persev. | d/pMBI-md | 4 |
| Apa.+LS/E+AEB | aMBI-md | 4 |
| Disin.+Persev. | d/pMBI-md | 4 |
| Apa.+Persev.+AEB | ad/pMBI-md | 3 |
| Disin.+Persev.+AEB | d/pMBI-md | 3 |
| Apa.+Disin.+LS/E+Persev.+AEB | ad/pMBI-md | 3 |
| Persev.+AEB | d/pMBI-md | 3 |
| LS/E+AEB | uMBI-md | 2 |
| Apa.+Psychosis | psyMBI-md | 2 |
| AEB+Psychosis | psyMBI-md | 2 |
| LS/E+Persev.+AEB | d/pMBI-md | 1 |
| LS/E+Psychosis | psyMBI-md | 1 |
| Apa.+LS/E+Persev.+Psychosis | psyMBI-md | 1 |
| Apa.+Disin.+LS/E+AEB | ad/pMBI-md | 1 |
| Apa.+Disin.+LS/E+Psychosis | psyMBI-md | 1 |
| Apa.+Disin.+AEB | ad/pMBI-md | 1 |
| Apa.+Disin.+Persev.+AEB | ad/pMBI-md | 1 |
| Disin.+LS/E+Persev. | d/pMBI-md | 1 |
| Disin.+LS/E+Persev.+AEB | d/pMBI-md | 1 |
| Disin.+AEB+Psychosis | psyMBI-md | 1 |
| AEB+Persev. | d/pMBI-md | 1 |
| Apa.+Disin.+LS/E+Persev.+AEB+Psychosis | psyMBI-md | 1 |

**Notes.** MBI=mild behavioural impairment; Apa.=apathy; Disin.=disinhibition; Persev.=perseveration; AEB=altered eating behaviour; LS/E=loss of sympathy/empathy; MBI=mild behavioural impairment; aMBI-sd=apathetic MBI – single-domain; aMBI-md=apathetic MBI – multiple-domain; ad/pMBI-md=apathetic-disinihibited/perseverative MBI – multiple-domain; d/pMBI-md=disinihibited/perseverative MBI – multiple-domain; uMBI-md=unspecified MBI – multiple-domain; psyMBI-sd=psychotic MBI – single-domain; psyMBI-md=psychotic MBI – multiple-domain; uMBI-md=unspecified MBI – multiple domain.
